# Molecular and Genetic Interactions between CCN2 and CCN3 behind Their Yin–Yang Collaboration

**DOI:** 10.3390/ijms23115887

**Published:** 2022-05-24

**Authors:** Satoshi Kubota, Kazumi Kawata, Takako Hattori, Takashi Nishida

**Affiliations:** Department of Biochemistry and Molecular Dentistry, Graduate School of Medicine, Dentistry and Pharmaceutical Sciences, Okayama University, Okayama 700-8525, Japan; ph092dix@okayama-u.ac.jp (K.K.); hattorit@cc.okayama-u.ac.jp (T.H.); tnishida@md.okayama-u.ac.jp (T.N.)

**Keywords:** cellular communication network factor, CCN2, CCN3, cartilage, fibrosis, glycolysis

## Abstract

Cellular communication network factor (CCN) 2 and 3 are the members of the CCN family that conduct the harmonized development of a variety of tissues and organs under interaction with multiple biomolecules in the microenvironment. Despite their striking structural similarities, these two members show contrastive molecular functions as well as temporospatial emergence in living tissues. Typically, CCN2 promotes cell growth, whereas CCN3 restrains it. Where CCN2 is produced, CCN3 disappears. Nevertheless, these two proteins collaborate together to execute their mission in a yin–yang fashion. The apparent functional counteractions of CCN2 and CCN3 can be ascribed to their direct molecular interaction and interference over the cofactors that are shared by the two. Recent studies have revealed the mutual negative regulation systems between CCN2 and CCN3. Moreover, the simultaneous and bidirectional regulatory system of CCN2 and CCN3 is also being clarified. It is of particular note that these regulations were found to be closely associated with glycolysis, a fundamental procedure of energy metabolism. Here, the molecular interplay and metabolic gene regulation that enable the yin–yang collaboration of CCN2 and CCN3 typically found in cartilage development/regeneration and fibrosis are described.

## 1. CCN2 and CCN3 in CCN Family

Cellular communication network factors 2 and 3 (CCN2 and CCN3) are two out of three founding members of the CCN family, which comprises six members in mammals. Initially, CCN2 was designated connective tissue growth factor (CTGF), owing to its mitogenic activity in fibroblasts [1], whereas the original name of CCN3 was nephroblastoma-overexpressed (NOV), since it was found to be overexpressed in a truncated form in nephroblastomas [2]. Added with the first founding member, cysteine-rich 61 (CYR61) [3], which is now generally recognized as CCN1, the CCN family was born as the acronym of these classical members [4,5], based on their striking structural similarities. It was a fortunate that the family name was not given in memory of CTGF, an impressive name representing a molecular function, as most CCN family members, particularly CCN3, turned out to be anything but growth factors [6,7,8]. After the establishment of this family, three additional members were added, completing the present form of this family of six members. Since each additional member was discovered by several independent research groups [9,10,11], a few different names were given to a single protein, which caused significant confusion. Among these names, Wnt-induced secretory protein (WISP) 1, 2 and 3 were preferred by a significant number of researchers, probably because these names represent a simple property of these proteins, although their official names were proposed as CCN4, 5 and 6 by the International CCN Society (ICCNS).

Along with the advance in CCN family research, it has gradually been clarified that CCN family members act as integrative regulators of extracellular signaling under interaction with a number of biomolecules in the microenvironment, yielding highly context-dependent biological outcomes [12,13,14,15,16,17]. Therefore, in 2018, the Human Genome Organization (HUGO) Gene Nomenclature Committee approved the new CCN nomenclature proposed by the ICCNS Scientific Committee [18,19]. According to this smart proposal, the CCN family was redefined as the cellular communication network factor family, which best represents the functionality of the family members, instead of an acronym without a specific meaning. Therefore, using this terminology is strongly encouraged now.

**Figure 1:**
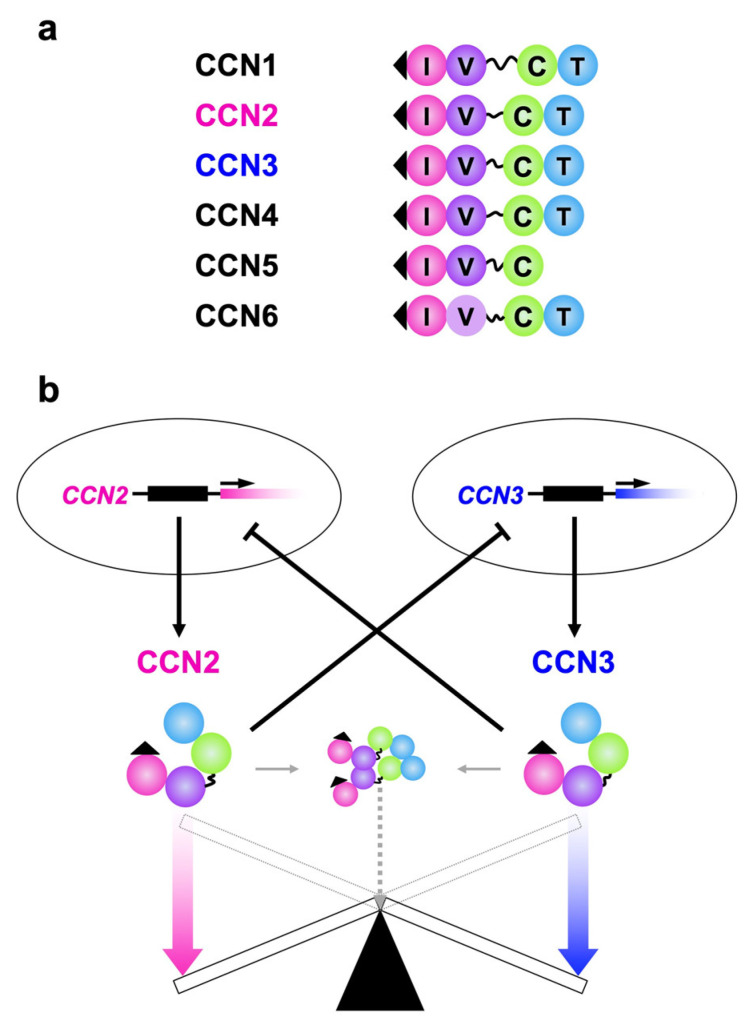
(**a**) Structures of all of the CCN family members. I, V, T and C indicate insulin-like growth factor binding protein-like (IGFBP), von Willebrand factor type C repeat (VWC), thrombospondin I type I repeat (TSP1) and C-terminal cystine-knot (CT) modules, respectively. A series of 38 cysteine residues in these modules are strictly conserved, whereas CCN6 lacks four of them in VWC. Signal peptides for secretion on the IGFBP modules (solid triangles) and hinges between VWC and TSP are also illustrated. The hinge domain is highly variable among the members, which is particularly long in CCN1. (**b**) The yin–yang collaboration of CCN2 and CCN3. The gene products with apparently opposite functions negatively regulate the expression of the other, while conferring opposing biological effects.

As is found in the case of other family members, CCN2 and CCN3 are commonly characterized by the retention of a tetra modular structure with signal peptides for secretion ahead and a hinge domain between the second and third modules from the amino termini. One exceptional member is CCN5 of tri-modular structure, which lacks the C-terminal module (Figure 1a). By using these four (or three for CCN5) modules—namely, insulin-like growth-factor binding-protein-like (IGFBP), von Willebrand factor type C repeat (VWC), thrombospondin 1 type 1 repeat (TSP1) and C-terminal cystine knot (CT) modules—both proteins bind to growth factors, extracellular matrix components and cell surface receptors, some of which interact with both CCN2 and CCN3 [12,13,14,15,16,17]. CCN2, which is probably the best investigated CCN family member, is known to directly bind to vascular endothelial growth factor (VEGF) [20], fibroblast growth factor (FGF) 1, FGF-2 [21,22], bone morphogenetic protein (BMP) 2 [23], BMP-4 [24], transforming growth factor (TGF) β [24], insulin-like growth factor (IGF) 1 and IGF-2 [25] as a partner of growth factors. As a matricellular protein, CCN2 has an affinity with fibronectin [26], aggrecan [27] and heparan sulfate proteoglycans such as perlecan [28]. As cell-surface receptors that accept CCN2 as a ligand, integrins with a variety of combinations of α and β subunits [29,30,31,32], FGF receptor (FGFR) 1, FGFR-2, FGFR-3 [22,33], epidermal growth factor receptor (EGFR) [34,35], tropomyosin receptor kinase A (TrkA) [36], low-density lipoprotein receptor-related protein (LRP) 1 [37], LRP-6 [38], receptor activator of nuclear factor kappa B (RANK) and its decoy receptor, osteoprotegerin [39] are known. Association with extracellular signaling modulators that include Wnt inhibitory factor (WIF) 1 [40] and slit guidance ligand (SLIT) [41] 3 was reported as well. Moreover, direct interaction between CCN2 and CCN3 was confirmed (Figure 1, in the middle) [42], the details of which are introduced later on. To date, fewer molecular counterparts have been identified for CCN3. This is assumed to be not because CCN3 is less interactive, but because it is relatively less investigated. Currently, fibulin 1c [43], Notch 1 [44], periostin [45] as well as BMP-2 and integrins [15] that are common between the two, were found as CCN3 counterparts. Extensive studies would discover as many cofactors for CCN3 as those found for CCN2 in the future.

Despite such striking structural and behavioral similarities, these two family members appear to functionally counteract each other and negatively regulate the gene expression of the other in a yin–yang manner (Figure 1b) [46,47,48]. Namely, investigation with kidney mesangial cells indicated that CCN3 markedly downregulated CCN2 production, leading to the blockade of the accumulation of extracellular matrix caused by CCN2. Moreover, TGF-β treatment reduced *CCN3* expression, while inducing *CCN2* in those cells and human dermal fibroblasts. The opposing biological effects of CCN2 and CCN3 and mutual repressive regulation were also observed in chondrocytes [7]. In this review, recent findings regarding the mechanism and biological significance of the intimate interactions between CCN2 and CCN3 are introduced in a brief but comprehensive fashion.

## 2. Yin–Yang Actions of CCN2 and CCN3

Usually, the molecular functions of CCN2 and CCN3 appear to be opposing. This is most typically represented by the effect of these CCN family proteins on cell proliferation in vitro. As exactly suggested in the original name of CCN2, early studies revealed its mitogenic activity on vascular endothelial cells, chondrocytes, osteoblasts, periodontal ligament cells and tumor cells, as well as fibroblasts [49,50,51,52,53,54,55,56]. In contrast, the original name of CCN3 is based on the positive relationship between the tumor development and the truncated form of this protein [2], suggesting a mitogenic activity of the mutant, which at the same time implies either a distinct, or less robust effect of the full length on cell proliferation. In accordance with this expectation, subsequent studies revealed the antiproliferative effect of CCN3 on chondrocytes, osteoblasts, vascular smooth muscle cells and tumor cells [6,7,57,58,59,60,61].

However, CCN2 and CCN3 occasionally exert comparable functionalities under particular biological context. Typically, both CCN2 and CCN3 promote cell adhesion and migration [62,63,64], which constitutes the proangiogenic property that is shared by these apparently contrastive members [65,66]. It should be noted that CCN1, the other founding member of the CCN family, also acts as a proangiogenic factor [67,68]. As stated in the next section, this common functionality is enabled by the interaction of CCN1, CCN2 and CCN3 with the same molecular counterparts, integrins [14,15,16,29,66,67]. As such, despite their prominent counter action with regard to cell proliferation, one of the most fundamental cellular events, CCN2 could not simply be a foe of CCN3 in living bodies, and vice versa.

These four-handed friendly proteins are surrounded not only by their common molecular friends, but also by a number of proper counterparts in microenvironment. By shaking hands with these colleagues, these versatile proteins execute multiple missions in tissues and organs. Indeed, CCN2 plays a critical role in the development and maintenance of the olfactory bulb in the central nervous system [69], skeletal system [70,71], neuromuscular junctions [72], pancreas [73], hair and teeth [74,75]. Interestingly, CCN3 was found to be critically involved in biological states and locations distinct from those where CCN2 is highly active. In fact, CCN3 promotes hematopoietic stem-cell renewal and recruitment [76,77,78], where no significant contribution of CCN2 has been suggested. Where CCN2 is present and/or active, CCN3 is not, which is in part enabled by the genetic interaction between CCN2 and CCN3. Therefore, CCN2 and CCN3 are constructing and maintaining life by playing their proper roles in a yin–yang fashion, under mutual molecular and genetic interactions, which is reminiscent of the roles of male and female, or darkness and lightness in the world.

## 3. Molecular Interaction between CCN2 and CCN3

It is generally recognized that two biomolecules with similar functions require the same cofactors at work. On the other hand, as is observed in the case of antagonists, counteractive functions of two molecules can be sometimes ascribed to competitive binding to the common counterpart. Therefore, sharing the molecular counterparts confers the indirect molecular interaction in between, which forms a basis of integrated molecular functionality of CCN2 and CCN3. In fact, CCN2 and CCN3 binds to BMPs [23,24,79,80,81], as well as the well-known adhesion receptors, integrins [14,15,16,29,30,31,32,65,66]. As already introduced in the last section, both CCN family members promote cell adhesion, migration and angiogenesis under interaction with these cell-surface molecules [62,65]. Other groups of the partners, BMPs, are widely known as morphogens, signaling molecules and bone inducing agents. Upon binding to BMPs, CCN2 and CCN3 diminish the BMP signaling in the cells [24,80]. However, the effects of CCN3 on osteogenesis are particularly controversial. Several reports indicated osteogenic potential, whereas others showed inhibitory effects [82,83,84], clearly representing the context dependence of the CCN-induced biological outcomes. The osteogenic effects observed in certain studies might be caused via CCN2- or CCN3-specific molecular partners that were present in the microenvironment under the experimental conditions employed therein. Mutual interference in the molecular actions between CCN2 and CCN3 via BMPs is also considerable if both are present.

In addition to such indirect interactions between CCN2 and CCN3 mediated by cofactors, it should be of particular note that CCN2 and CCN3 directly bind each other (Figure 1) [42]. Basically, production of CCN2 and CCN3 is regulated in a yin–yang fashion, avoiding their co-existence. Nevertheless, CCN2 and CCN3 coexist and encounter each other on specific occasions, where CCN2 and CCN3 are able to communicate directly. It should be noted that antagonizing effect of CCN2 on CCN3 by direct binding was observed in chondrocytes [42]. The direct CCN2–CCN3 interaction can be a molecular switch for forwarding the cells to the next stage during biological processes, including endochondral ossification, which is detailed in Section 5.

## 4. CCN2-CCN3 Genetic Interaction and Its Mechanism

**Figure 2:**
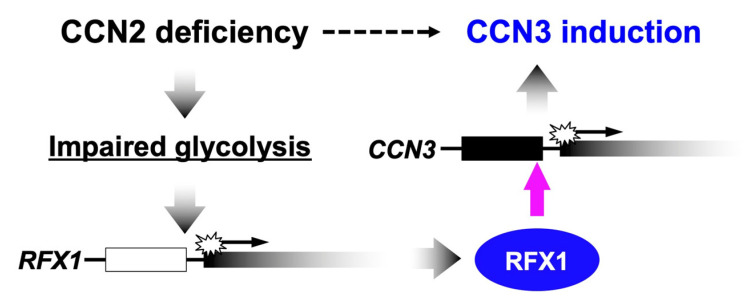
Mechanism of the negative regulation of *CCN3* by CCN2 in chondrocytes. A transcriptional factor regulatory factor binding to the X-box (RFX1) mediates this metabolic gene regulation.

Unlike the molecular functionalities, temporospatial emergence of CCN2 and CCN3 in a yin–yang fashion does not result from their molecular interactions. Where and when CCN3 is, CCN2 may not be produced, and vice versa. In order to firmly support this situation, a mutual negative regulatory system is quite effective, and is actually furnished in our cells. Several reports in the past indicated that overexpression or exogenous addition of either CCN2 or CCN3 attenuated the gene expression of the other in several types of cells [7,46]. Consistent with these findings, highly elevated expression of CCN3 was observed in murine *Ccn2*-null chondrocytes [7]. However, the molecular mechanisms of these regulatory systems remained totally unclear for a long time. Recently, a genetic mechanism that mediates the negative regulation of CCN3 by CCN2 was uncovered in chondrocytes. In those cells, CCN2 depletion caused severe ATP deficiency. Metabolomic and transcriptomic analyses revealed that metabolic intermediates of glycolysis and the gene expression of a series of enzymes that catalyze the reactions therein were both attenuated in *Ccn2*-null chondrocytes. In contrast, no appreciable changes were observed in the mitochondrial membrane potential in those cells. These findings strongly indicate that CCN2 deficiency leads to impaired glycolysis. [85,86]. In relation to this metabolic impact of CCN2 deficiency, recent studies revealed that CCN3 expression was strongly enhanced at a transcriptional level by impaired glycolysis [87]. Indeed, CCN3 expression was markedly induced by biochemical inhibition of two different enzymes involved in the glycolytic pathways, as well as by glucose starvation in chondrocytic cells. Subsequent study identified regulatory factor binding to the X-box (RFX) 1 as a transcription factor that binds to the enhancer in the CCN3 proximal promoter region and mediates this metabolic gene regulation (Figure 2) [88]. Therefore, in chondrocytes, glucose metabolism is a central mediator of the repressive regulation of *CCN3* by CCN2. However, in contrast with the negative regulatory machinery of CCN3 by CCN2, the mechanism of how CCN3 represses CCN2 is still poorly understood.

Regarding the metabolic regulation of CCN family members, it is also of note that CCN2 expression is repressed by glycolysis inhibitors and glucose starvation that contrarily induces CCN3 in chondrocytes [87,88]. Collectively, glycolytic activity was found to upregulate CCN2 and downregulate CCN3 simultaneously in chondrocytes. Interestingly, a previous report indicated that stimulating astrocytes with TGF-β provoked simultaneous induction of CCN2 and repression of CCN3, while tumor necrosis factor (TNF) α repressed CCN2 expression and enhanced CCN3 expression at the same time [89]. The simultaneous regulation of CCN2 and CCN3 towards opposite directions may not be explained by the mutual negative regulation among these genes and their products. However, considering the metabolic effects of these cytokines, a possible mechanistic view can be proposed based on the simultaneous regulation of CCN2 and CCN3 by glycolysis. Since TGF-β enhances glycolysis [90], CCN2 induction and CCN3 repression occur simultaneously after the stimulation. Contrarily, TNF-α triggers apoptosis that affects mitochondrial function [91], leading to impaired glycolysis. As such, *CCN2* and *CCN3* could be repressed and induced at the same time, respectively. Together, these findings suggest that metabolic regulation by glycolytic activity may constitute a central part of the regulatory machinery that enables the yin–yang emergence of CCN2 and CCN3. Most interestingly, the yin–yang emergence and function of these proteins are also observed in pancreatic islet that regulate systemic glucose metabolism [92]. CCN2 is produced during normal development of pancreatic islet to support cell proliferation and disappears after birth, whereas CCN3 is present in quiescent pancreatic β-cells and inhibits their proliferation. However, CCN3 is known to induce the development of obesity and diabetes [93], and this system appears deranged upon these pathological conditions. Although CCN2 is decreased along with adipocyte differentiation [94], its expression level is found to be increased in the fat tissues of the model mice of obesity and diabetes. Here, the systemic importance of the metabolic yin–yang regulation of CCN2 and CCN3 is implicated.

In addition to the metabolic regulation, it is widely recognized that CCN2 is directly regulated by TGF-β through TGF-β-associated kinase, mitogen-activated protein kinase (MAPK) -extracellular signal-regulated kinase (ERK) kinase (MEK) and specificity protein (SP) 1/SP3, as well as the canonical Smad pathway [48,95]. Involvement of other regulatory pathways is also suspected in the *CCN3*-part of the yin–yang regulation. To date, negative regulation of *CCN3* by TGF-β was reported in nucleus pulposus cells, which was shown to be Smad independent [96].

## 5. CCN2-CCN3 Collaboration in Cartilage

**Figure 3:**
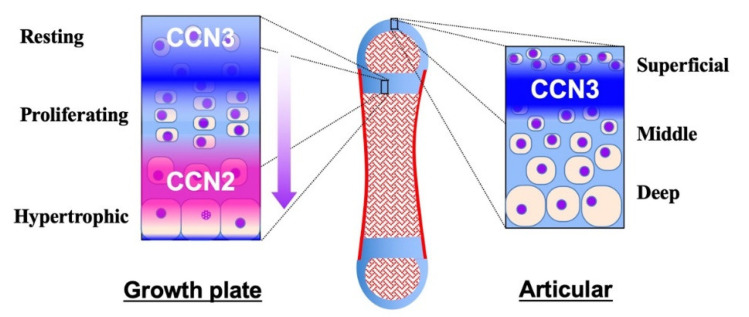
Distribution of CCN2 and CCN3 in the growth plate (**left**) and the articular cartilage (**right**) in a long bone (**middle**). Cartilage and bone tissues are shown in light blue and red, respectively. Distinct, but partly overlapping distribution of CCN2 and CCN3 is illustrated with gradation. Zones in the growth plate and articular cartilage are indicated. The arrow at the right of the growth plate indicates the direction of chondrocytic differentiation and bone growth.

The yin–yang behavior of CCN2 and CCN3 during tissue development, maintenance and regeneration is typically found in the skeletal system. Roughly speaking, CCN2 promotes these processes, whereas CCN3 acts as a regulator or repressor therein, emerging and disappearing in turn. Most bones constituting our skeleton are formed through an integrated process called endochondral ossification. In this process, the bone anlage is first formed as cartilage. Thereafter, chondrocytes grow bones as cartilage, following a series of procedures which develop the growth plate therein (Figure 3, left panel). At the earliest stage, CCN3 is expressed in this avascular prototype of the bone with its higher expression levels inside [97]. Owing to the poor nutrition supply, CCN3 expression at this stage is the highest in the central region. CCN3 exerts angiogenic activity to lead blood vessels inside, which results in ossification center formation, the beginning of endochondral ossification. The CCN3 accumulated at the ossification center stays with hypertrophic chondrocytes around [98], which may assist those cells in ceasing proliferation. In the regions furthest from the vascular invasion, CCN3 expression in immature chondrocytes is retained at high levels, keeping the cells quiescent as resting chondrocytes. From this region towards the ossification center, CCN3 expression gradually diminishes, allowing them to proliferate as proliferating chondrocytes [7]. As CCN3 decreases, CCN2 increases in turn, because of the improved glycolysis supported by the nutrition infiltrating from the blood vessels. CCN2 accelerates both proliferation and differentiation as a communication network factor to grow the cartilage, and its production reaches a peak in the highly differentiated pre-hypertrophic chondrocytes, where CCN3 is not actively produced [7]. Finally, around the site of vascular invasion, terminally differentiated hypertrophic chondrocytes disappear, forming matrix vesicles, on which osteoblasts accumulate mineralized crystals to build bone tissues [97]. In spite of their differential gene expression, CCN2 and CCN3 encounter each other during the proliferative stage [7]. It is suspected that the direct binding of CCN2 to CCN3 [42] may antagonize the antiproliferative effect of CCN3, whereas the differentiating effect of CCN2 may be also attenuated, which would promote chondrocytes from resting to proliferative stages.

After the completion of bone growth, the growth plate disappears in humans, leaving articular cartilage at both ends. Articular cartilage stays until the end of the life, supporting the locomotive activities, unless damaged or offended by osteoarthritis. In normal articular cartilage, CCN2 is barely detected [99], while CCN3 is produced by a limited population of articular chondrocytes located beneath the superficial layer of the cartilage (Figure 3, right panel) [96], which is far from the nutrition source, bone marrow or synovial fluid. CCN3 is induced therein by nutrition shortage and contributes to the retention of the quiescence and cellular stemness of chondrocytes around it [100]. However, along with aging, this restricted mode of CCN3 production in articular cartilage gradually changes. Immunohistochemical evaluation of mouse articular cartilage revealed that CCN3 was produced only by a limited number of the cells beneath the superficial layer of articular cartilage in young mice at one or two months after birth. In contrast, in aged mice at 7 months after birth, the majority of the chondrocytes in the superficial layer were found to produce CCN3. This age-related accumulation of CCN3 in articular cartilage was confirmed by biochemical analysis of corresponding animals. Furthermore, significant positive correlation was found between CCN3 expression levels and ages in human articular chondrocytes [8]. Contrarily, only a few chondrocytes were found to be producing CCN2 in aged mice through immunohistochemical analysis [8]. It is highly suspected that CCN3 induced by aging may repress the CCN2 production therein. The CCN3-dominant microenvironment of aged articular cartilage is supposed to be an adaptive response to the locomotive and nutrition conditions of senior individuals, but can be a risk factor for developing osteoarthritis. As a matter of fact, cartilage-specific overexpression of CCN2 in mice adds resistance to age-related osteoarthritis development [99].

**Figure 4:**
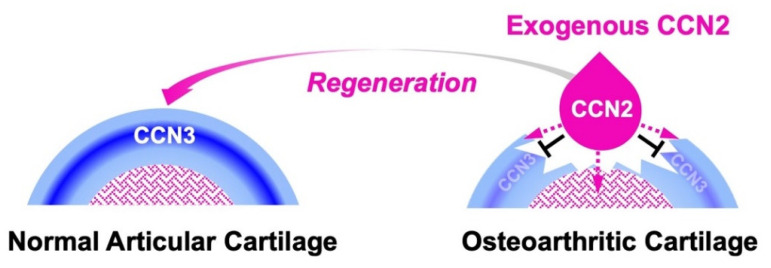
Molecular action of exogenous CCN2 applied to regenerate damaged articular cartilage. Under healthy conditions, CCN3 is produced immediately beneath the superficial zone to maintain the stemness of chondrocytes (**left**). In the articular cartilage degenerated by osteoarthritis (**right**), exogenously applied CCN2 (droplet in pink) releases the articular chondrocytes in the superficial zone from stemness by inhibiting the production of CCN3 and forwards them to cartilage regeneration (black T-bars). CCN2 also stimulates chondrocyte progenitors in bone marrow towards cartilage regeneration (dotted lines in pink).

In general, damaged articular cartilage hardly regenerates without therapeutic intervention. Nevertheless, CCN2 was found to promote the regeneration of damaged articular cartilage in rat osteoarthritis models [101,102]. Namely, exogenously applied CCN2 promotes the proliferation and differentiation of the remaining cells; furthermore, this CCN family member strongly represses the production of CCN3 through the metabolic regulatory system introduced in a previous section (Figure 4) [7,88]. As a consequence of this yin–yang regulation, articular chondrocytes are released from the quiescent stem-cell stage and are engaged in cartilage reconstruction encouraged by the molecular function of CCN2. As such, this mutual regulatory system is also useful for maintaining and recovering the integrity of the permanent cartilage in synovial joints.

## 6. CCN2-CCN3 Interaction in Fibrosis and Inflammation

**Figure 5:**
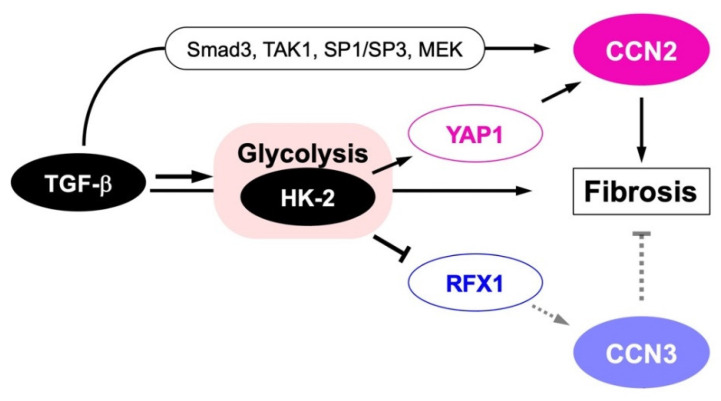
Metabolic yin–yang regulation of CCN2 and CCN3 during fibrotic disorder development. TGF-β signal stimulates CCN2 both directly through specific transcription factors, including Smad3, TGF-β activated kinase 1 (TAK1), specificity protein (SP) 1/SP3 and mitogen-activated protein kinase (MAPK) -extracellular signal regulated kinase (ERK) kinase (MEK), and indirectly through the activation of glycolysis by accumulating hexokinase (HK)-2, which is mediated by yes-associated protein (YAP) 1. Glycolysis activation by TGF-β through HK-2 contrarily represses RFX1 expression, resulting in the repression of antifibrotic CCN3 production. Smad-independent negative regulation of CCN3 by TGF-β observed in nucleus pulposus cells could occur through this metabolic pathway. Finally, CCN2 protein promotes fibrosis in collaboration with TGF-β.

Since the discovery of CCN2, this protein has been regarded as a key player of fibrotic disorders in a variety of tissues and organs. A typical example is scleroderma, which is an intractable skin disease characterized by progressive fibrosis in skin. From early CCN2 research, the involvement of this factor has attracted the interest of the researchers in this field [103]. CCN2 was found to be highly expressed in the region of this disease [103,104], and the CCN2 levels in blood and skin correlated to the disease severality [105,106]. Accordingly, neutralizing antibody against CCN2 efficiently ameliorated it in an animal model in vivo [107]. Similarly in gingival fibrosis induced by nicotine exposure, CCN2 mediates this local effect of nicotine [108]. Gingival overgrowth with fibrosis occurs by medications with phenytoin [109,110] and nifedipine [109,110,111], in which CCN2 is involved as well. Fibrosis is also induced by bleomycin in lung, in which collagen overproduction is conducted by CCN2 [112,113]. Indeed, an anti-CCN2 antibody was shown to be effective to slow down the progression of idiopathic pulmonary fibrosis [113]. In addition to lung, the contribution of CCN2 to the development of fibrosis is known in the heart [114], liver, kidney [106,115], pancreas [116] and skeletal muscles [117]. As such, diagnostic values of blood and even urine CCN2 levels as biomarkers in these fibrotic diseases are discussed [106]. Not only typical fibrotic disorders, but also several diseases accompanied by fibrotic changes in tissues, such as atherosclerosis [118], proliferative retinopathy [119] and neuromuscular diseases, such as several types of muscular dystrophy, muscle denervation, amyotrophic lateral sclerosis and spinal muscular atrophy [117] are known to be related to CCN2. Therefore, CCN2 has been regarded as a highly potential target for the development of drugs that could be used in all of these fibrotic diseases [120,121,122,123]. Considering the counteracting molecular function of CCN3 against CCN2, it was reasonable that the yin–yang emergence and function of CCN2 and CCN3 were initially noted in the context of fibrosis [46]. It was widely recognized that CCN2 is involved in renal fibrosis [124], which has been confirmed by several later studies as well [46,125]. As renal fibrosis is a terminal pathological situation of diabetes, antagonists of CCN2 were expected as therapeutic agents of this severe and common complication of diabetes in humans. Along with the advance in relevant research, investigation using mesangial cells and dermal fibroblasts revealed that TGF-β regulated CCN2 and CCN3 in a bipartite and opposite manner, representing their yin–yang regulation [46,48]. Moreover, CCN3 overexpression strongly repressed CCN2 and type I collagen production, a hallmark of fibrosis, indicating the negative regulation of CCN2 and antifibrotic action of CCN3. Gene regulation and functional counteraction were suggested to be critical in maintaining the healthy renal state, as it was found collapsed at the late stage of diabetic nephropathy. The fact that either CCN2 or CCN3 deficiency predisposes aortic aneurysm is also notable [126,127]. Comparable findings were also obtained by the experiments with a non-alcoholic steatohepatitis model [128], embryonic fibroblasts [129], astrocytes and hepatic stellate cells [130], suggesting the universal role of this yin–yang system in the maintenance of extracellular matrix metabolism throughout the body. Therefore, the dysregulation or collapse of this system may well incur fibrosis, potentially in any tissue or organ. Mechanistically, as observed in the regulation of CCN3 by CCN2, TGF-β-induced glycolysis through hexokinase (HK) 2 [131] seems to provoke CCN2 production via yes-associated protein (YAP) 1 [48,130], while silencing CCN3 via RFX1 [87,88] (Figure 5).

Regarding fibrosis, the contributions of the other CCN family members are also highly suspected. CCN1 is regulated by the profibrotic TGF-β in a manner similar to that of CCN2 [48]. Upon the repression of fibrotic response by CCN3 overexpression, CCN4 expression was shown to be even better repressed than CCN2 [82]. CCN5 was reported to act as an antifibrotic agent during the development of cardiac hypertrophy with fibrosis [132,133]. Lastly, a report once indicated the profibrotic role of CCN6 in pulmonary fibrosis [134]. However, evidence that indicates a more distinct yin–yang action between the other CCN family members than that between CCN2 and CCN3 has not been presented until now.

Fibrosis is the outcome of the prolonged and dysregulated tissue reconstruction after inflammatory responses, and thus can be regarded as a chronic disorder occurring at the final stage of inflammation. In this context, it should also be noted that CCN2 and CCN3 are occasionally regulated in a yin–yang fashion by typical mediators of inflammation other than TGF-β. As already introduced in a previous section, TNF-α represses *CCN2* expression, while activating *CCN3* in astrocytes [89]. This CCN3 activation is suspected to be mediated by NF-κB, since an NF-κB inhibitor repressed CCN3 expression in A549 cells [135]. However, in A549 cells, CCN3 expression was induced by TGF-β as well, suggesting that the yin–yang regulation system is not retained in these cells. Similar yin–yang regulation was observed by the stimulation with IL-1β, another representative inflammatory cytokine [136,137]. On the other hand, once produced upon inflammation, these CCN family members do not necessarily behave in this manner. Indeed, both CCN2 and CCN3 were reported to induce the same chemokine, monocyte chemotactic protein (MCP) 1 [89], suggesting the complex involvement of CCN family in inflammatory responses.

## 7. CCN2-CCN3 Interplay in Malignancies

In addition to fibrosis, a number of studies have indicated the profound involvement of CCN2 and CCN3 in human malignancies [138]. In some cases, the yin–yang emergence of CCN2 and CCN3 is retained, whereas the CCN2/CCN3 bidirectional regulation system appears to be collapsed in the others [87,139,140,141,142]. Because of the context dependence of these proteins, both CCN family member can be promotors and suppressors of tumors, depending upon what kind of partners are present in the tumor microenvironment. As a general tendency, upregulation of CCN2 and downregulation of CCN3 are observed in colorectal cancer cases [138]. In these cancers with common origin, a negative relationship between severeness and CCN3 expression levels was shown [139], suggesting that it results from the antiproliferative function of CCN3, but the pathological role of CCN2 still remains inconclusive. On the other hand, CCN2 and CCN3 are both reported to be elevated and regarded as promoting factors of malignant phenotype in liver cancers, which implies that the yin–yang regulatory system may be disabled therein. However, when the expression levels of CCN2 and CCN3 in the same cases of hepatocellular carcinoma were compared, induction and reduction of the expression of CCN3 and CCN2, respectively, were observed, indicating the retention of the yin–yang regulatory system in these particular cases [140]. In contrast, in another study with a cohort with 122 breast cancer cases, both CCN2 and CCN3 levels were rather repressed in aggressive breast cancer tissues compared to those in the normal ones [141]. It is widely recognized most malignant tumor cells undergo metabolic reprogramming biased towards glycolysis, which is called the Warburg effect [142]. Nowadays, a number of driving factors, such as hypoxia-inducible factor 1, oncogene products, mitogenic signaling pathways, glucose and lactose transporters and glycolytic enzymes, are shown to be involved in the development of this reprogramming of energy metabolism [142]. Deficiency in AMP-activated protein kinase pathway and tumor suppressor genes may promote the establishment of the Warburg effect as well [143,144]. Since glycolysis constitutes a central machinery of the bidirectional CCN2/CCN3 regulation, dysregulated glycolysis may well destroy the delicately balanced yin–yang regulation. For example, CCN2 expression could even be enhanced by impaired glycolysis in breast cancer cells [145]. Therefore, whether this system is retained or not may represent the metabolic status of malignancies, which may reflect the property of tumor cells and even the prognosis of the patients.

## 8. Conclusions

During physiological tissue development and regeneration, the yin–yang collaboration of CCN2 and CCN3 plays a critical role, as clearly observed in skeletal development and maintenance processes. Unfortunately, this integrated regulatory system is exploited in the process of fibrosis development. However, as far as this bidirectional regulatory system is properly retained, we may be able to revert the fibrotic lesions to normal ones by manipulating the same system. This idea may provide a clue for development of a new therapeutic strategy to combat intractable fibrotic disorders. Until now, CCN2 has already been regarded as a major target, as well as a clinical marker, in combating fibrosis. As a matter of fact, a neutralizing antibody against human CCN2 was developed and subjected to clinical trials for the treatment of idiopathic pulmonary fibrosis [146], muscular dystrophy [121], diabetes [147] and locally advanced pancreatic cancer cases [148]. In contrast, therapeutic utility of CCN3 as an antifibrotic or anticancer agent was proposed, but has not been forwarded to clinical trials to our knowledge. CCN3 can be a therapeutic agent as effective as an anti-CCN2 antibody, if the yin–yang regulatory system is retained. Although it may not be effective for the treatment of cancers with yin–yang regulatory system deficiency, development of CCN3-based therapeutics for fibrosis and related diseases is highly expected in the near future.
